# High-harmonic spectroscopy of quantum phase transitions in a high-Tc superconductor

**DOI:** 10.1073/pnas.2207766119

**Published:** 2022-09-26

**Authors:** Jordi Alcalà, Utso Bhattacharya, Jens Biegert, Marcelo Ciappina, Ugaitz Elu, Tobias Graß, Piotr T. Grochowski, Maciej Lewenstein, Anna Palau, Themistoklis P. H. Sidiropoulos, Tobias Steinle, Igor Tyulnev

**Affiliations:** ^a^CMAB-CSIC, Institut de Ciència de Materials de Barcelona, Consejo Superior de Investigaciones Científicas, Universitat Autònoma de Barcelona, 08193 Bellaterra, Spain;; ^b^ICFO, Institut de Ciencies Fotoniques, The Barcelona Institute of Science and Technology, 08860 Castelldefels, Spain;; ^c^ICREA, 08010 Barcelona, Spain;; ^d^Physics Program, Guangdong Technion–Israel Institute of Technology, Shantou 515063, China;; ^e^Technion–Israel Institute of Technology, 32000 Haifa, Israel;; ^f^Center for Theoretical Physics, Polish Academy of Sciences, 02-668 Warsaw, Poland;; ^g^Institute for Quantum Optics and Quantum Information, Austrian Academy of Sciences, A-6020 Innsbruck, Austria;; ^h^Institute for Theoretical Physics, University of Innsbruck, A-6020 Innsbruck, Austria

**Keywords:** superconductivity, material science, quantum materials, spectroscopy, attoscience

## Abstract

We report on the nonlinear optical signatures of quantum phase transitions in the high-temperature superconductor YBCO, observed through high harmonic generation. While the linear optical response of the material is largely unchanged when cooling across the phase transitions, the nonlinear optical response sensitively imprints two critical points, one at the critical temperature of the cuprate with the exponential growth of the surface harmonic yield in the superconducting phase and another critical point, which marks the transition from strange metal to pseudogap phase. To reveal the underlying microscopic quantum dynamics, a strong-field quasi-Hubbard model was developed, which describes the measured optical response dependent on the formation of Cooper pairs. Further, the theory provides insight into the carrier scattering dynamics and allows us to differentiate between the superconducting, pseudogap, and strange metal phases. The direct connection between nonlinear optical response and microscopic dynamics provides a powerful methodology to study quantum phase transitions in correlated materials. Further implications are light wave control over intricate quantum phases, light–matter hybrids, and application for optical quantum computing.

## Significance

High-temperature superconductors and quantum phase transitions are technology building blocks to address standing problems from energy storage and transport to quantum sensing and optical quantum computing. Yet, the detection of different quantum states of the material and our understanding of the underlying physics are incomplete. We show high-harmonic spectroscopy (HHS) is an ultrasensitive probe of the dynamic evolution of quantum phase transitions. HHS identifies all transitions between quantum phases in the strongly correlated material YBCO, which are very difficult to detect even by transport measurements. A strong-field quasi-Hubbard framework provides the theoretical connection between measurement and the underlying microscopic dynamics.

Attosecond technology (1), specifically the process of high harmonic generation (HHG) (2–4), provides an all-optical probe of the microscopic dynamics of atoms, molecules, and solids. Shortly after the first observation of high harmonics in atoms, their generation was understood (4–6) as arising from electron recollision after strong field photoionization and excursion in the continuum. Since the harmonic signal strongly depends on the electron recollision angle and time, high-harmonic spectroscopy (HHS) is a sensitive nonlinear probe of microscopic electronic structure with atomic spatial and suboptical cycle temporal resolution. HHS of solids (7, 8), two-dimensional materials (9, 10), or nanostructured media (11, 12) differs from the gas phase since the optical field–driven electronic wave packet is delocalized over many lattice sites, the wave function depends on the lattice momentum, and a hole has to match the electron’s momentum for recombination to occur (13, 14). Recent experimental efforts extended HHS as nonperturbative probe to quantum materials (9, 10, 15, 16) and to topological insulators (17–19). There have also been several theoretical advances, which suggest using strong fields to probe the physics of Mott insulators (20, 21), alongside the possibility of optically modifying strongly correlated matter (22) and tracking optically induced phase transitions (23), with a recent experiment reported in ref. 24.

The sensitivity of HHS to the intricate microscopic details of carriers and lattice predestines HHS to investigate strong interactions and quantum correlations which lead to fascinating new states of matter such as superconductivity. The phase transition into a strongly correlated superconductive state is described by the spontaneous symmetry breaking of the U(1) redundancy when cooling below the critical temperature $T_{\text{c}}$ of the material. As we will show, HHS is a sensitive probe of the dynamic evolution of the superconducting phase transition since the formation of composite bosons by pairing two fermionic spin-1/2 particles (Cooper pairs) changes the distribution of charge carriers, and this sensitively registers in the high harmonic amplitudes and spectral distribution. Pictorially, this is described in *SI Appendix*, Fig. S1, by a three-step model, consisting of 1) interband excitation process, 2) intraband acceleration, and 3) interband recombination. Pairing below *T_c_* splits the bands by opening a superconducting gap Δ, and in the strongly correlated phase, the three processes of harmonic generation occur within the effective band structure for the Cooper pairs. We will also show that HHS can identify additional phase transitions between quantum phases in the strongly correlated material which are not accessible through the linear optical response, and they are difficult to detect with established methods such as superconducting quantum interference device (SQUID) magnetometry or four-probe transport measurements.

A conventional superconductor can be described by the Anderson–Higgs mechanism, which explains that an optical nonlinear response is due to a gapless phase mode (Nambu–Goldstein) and a gapped amplitude mode (Higgs) of the ordering parameter. In the simplest case, and depending on the strength and type of excitation, Boltzmann and Ginzburg–Landau theories (25, 26) predict a second-order response, which mixes with the excitation mode (27, 28), thus the generation of the third harmonic (29). Unconventional high-$T_{\text{c}}$ superconductors are of tremendous interest for a wide range of applications ranging from electronic devices and information processing devices to optical quantum computers and quantum simulators. However, due to their rich landscape of quantum phases and the difficulties of experimental methods to probe the microscopic dynamics, our understanding is still very limited.

Among the well-established methods, e.g., transport measurements (30) or magnetic torque measurements (31, 32), photoemission measurements such as angle-resolved photoemission spectroscopy (ARPES) (33, 34) provide direct access to a material’s microscopic carrier distribution and dynamics. The interpretation of such ARPES measurement is, however, complicated by the interpretation of the bulk spectral function and the assumption of independent electron emission despite measuring a strongly correlated electronic state of matter. These are central questions to access the nature of the multibody state, which call for further developments and powerful new tools to aid in the interpretation of the physical mechanism.

Therefore, the development of all-optical and ultrafast probes of the macroscopic dynamics inside such materials, which is compatible with existing methods, is highly desirable. To this aim, we apply HHS to investigate the transition between the different phases of the unconventional high-$T_{\text{c}}$ superconductor YBa_2_Cu_3_O${}_{7-d}$ (YBCO). We elucidate the connection between the measured optical spectra, the transition between strange metal and pseudogap phases, and the superconducting phase transition with a strong-field Hubbard model. The HHS measurement clearly shows a departure from the normal conducting phase with an increased formation of Cooper pairs upon cooling. The variation in harmonic orders is linked to phenomenological energy and phase relaxation times, which identify the transition to the fluctuation regime (35, 36), i.e., between the strange metal and pseudogap phases, and the sudden transition at $T_{\text{c}}$ into the superconducting phase. Unconventional superconductors, like YBCO, are material systems in which the formation of composite bosons out of paired fermions is mediated not by phonon exchange but by some other kind of energy exchange (37), for instance, due to spin fluctuations. Such systems present many standing fascinating questions. It is thus important to have new powerful experimental techniques like HHS that provide a fresh and alternate view of the problem.

## Results and Discussion

**Fig. 1.**
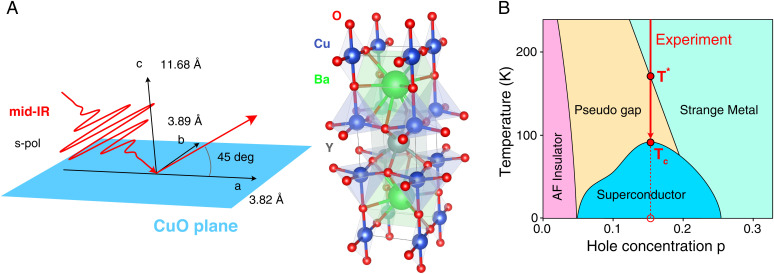
Experimental setup and YBCO properties: (*A*) Orthorhombic unit cell of YBCO together with the crystallographic axes. YBCO consists of alternating planes along the crystallographic c axis with order CuO–BaO–CuO_2_–Y–CuO_2_–BaO. Most relevant for superconductivity are the copper oxide planes. Also indicated is the experimental arrangement with linearly polarized mid-IR laser fields whose electric field vector lies along the copper oxide planes. (*B*) The phase diagram for YBCO. Based on the value of $T_{\text{c}}$ from the measurement, we determine a hole concentration of p ~ 0.15. Based on this value, we mark the transition between the different phases by the red arrow. In accord with measurement and theory, $T^{*}$ marks the transition between strange metal and pseudogap phases at 173 K.

YBCO is a copper oxide cuprate that crystallizes in an orthorhombic perovskite structure. The YBCO unit cell consists of two CuO_2_ planes which sandwich an oxygen-depleted CuO*_d_* plane (Fig. 1*A*). The CuO_2_ planes are central to superconductivity, interacting with oxygen-depleted CuO*_d_* chains which act as charge reservoirs (38–40), and specifically the interaction of electrons in the $2p_{x}$ and $2p_{y}$ orbitals of oxygen (O) ions and the $3d_{x^{2}-y^{2}}$ orbitals of copper (Cu) ions. Thus, YBCO is a quasi–two-dimensional superconducting system. As a result of the strong Coulomb repulsion by the Cu${}^{3+}$ ions, the $3d_{x^{2}-y^{2}}$ band splits into lower (LHB) and upper Hubbard bands (UHB) with a gap of up to 2 eV, and the density of states exhibits a peak at the top of the LHB. The transition to the SC phase is presently understood as hole-doping the oxygen 2*p* sites altering the exchange interaction between the copper spins which mediates the formation of Cooper pairs. Our measurement reveals two critical temperatures (discussed below) which allow determining the doping level of the material at p ~ 0.15. Fig. 1*B* depicts the phase diagram of YBCO, and based on the determined doping level for our YBCO sample, the red arrow indicates that our measurement transitions between the strange metal phase at room temperature into the pseudogap phase at $T^{*}$ and into the SC phase at $T_{\text{c}}$.

For our investigation, we measure high harmonics in reflection from 100-nm-thick films of YBCO which are mounted on a Joule–Thomson microrefrigerator. The superconducting properties of the YBCO films were characterized by inductive measurements, and a critical temperature of 88 K was obtained, indicating proper oxygen doping (*SI Appendix*, Fig. S2, and description of *SI Appendix*, YBCO sample fabrication and characterisation). Having confirmed the high quality of the YBCO films and the transition to the SC phase, we induce nonperturbative HHG with sub–band gap, ultrashort midinfrared (mid-IR) pulses at 3,200 nm (0.4 eV) from a mid-IR Optical Parametric Chirped Pulse Amplification (OPCPA) (41, 42). The linearly polarized 94-fs, mid-IR pulses are focused to a vacuum electric field strength up to 0.083 V/Å, and they intersect the material at 45^∘^ under s polarization. Due to the chosen reflection geometry with s polarization we avoid the generation of even-order surface harmonics, arising due to symmetry breaking at the interface, and detect the odd-order harmonics (HH3, HH5, and HH7) of the fundamental photon energy at 0.4 eV. This measurement geometry ensures the best possible signal-to-noise ratio as the fundamental’s energy is exclusively distributed among odd harmonics orders. To prevent oxidation of the samples and unwanted alteration of the superconductive properties, we conduct all experiments inside a vacuum chamber. We have checked that the 400-µm-thick CaF_2_ windows of the vacuum chamber do not contribute to the measured signal. The YBCO sample is cooled with the Joule Thomson refrigerator to a minimum temperature of 77 K, and we record the reflected radiation by imaging onto the entrance slit of a spectrograph for analysis.

**Fig. 2.**
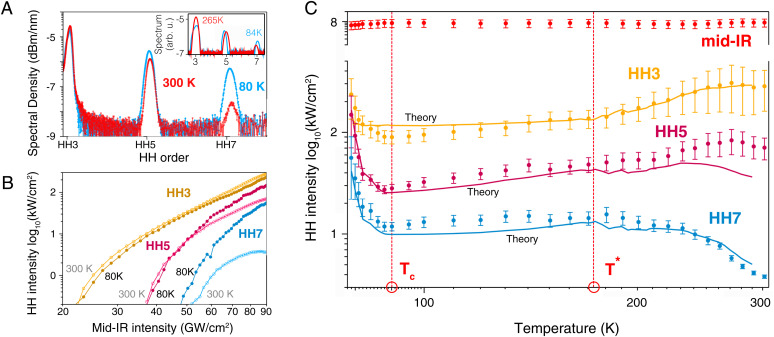
HHS of YBCO: (*A*) Harmonic spectra showing odd orders HH3, HH5, and HH7 for room temperature (red) and at $T_{\text{c}}=90$ K (blue) for a mid-IR field strength of 0.083 V/Å. Visible already is a blueshift of room-temperature harmonics with increasing harmonic order and relative to the harmonics measured at $T_{\text{c}}$. We observe a dramatic increase of HH7 amplitude upon cooling into the SC phase. The inlay shows the theoretical prediction of the spectrum, and the relative heights of the peaks match well the behavior of the experimental data. (*B*) The scaling of harmonic order with mid-IR peak intensity for measurements at room temperature and at $T_{\text{c}}$. Dark colors show data for the SC phase, whereas light colors indicate data at room temperature. (*C*) The reflected mid-IR field together with harmonics HH3, HH5, and HH7 as a function of temperature. These measurements are taken for a mid-IR field strength of 0.083 V/Å. Results from the strong-field quasi-Hubbard model are overlaid as solid lines. We observe a dramatic increase of HH7 amplitude upon cooling into the SC phase at 88 K. All harmonic orders show a clear turning point at the critical temperature $T_{\text{c}}$ and an exponential increase in amplitude. More subtle but still clearly discernible is another critical point $T^{*}$ at 173 K, marking the transition from strange metal to the pseudogap phase. The functional behavior is reproduced by the model. For completeness, we show the fundamental reflectivity also on a linear intensity scale in *SI Appendix*, Fig. S3.

Fig. 2*A* shows measured harmonic spectra at room temperature (red curve) and close to the critical temperature $T_{\text{c}}$ (blue curve). As expected, we record only odd-order harmonics. Clearly noticeable is a blueshift of room-temperature harmonics with increasing harmonic order and relative to the harmonics measured at $T_{\text{c}}$. Next, we record how HH3 (yellow), HH5 (red), and HH7 (blue) scale as a function of the mid-IR peak intensity, shown in Fig. 2*B*.

While it is expected that all harmonics increase for a higher peak intensity of the fundamental, we instead observe a surprising and dramatic increase for HH7 for the highest mid-IR peak intensity at 90 GW/cm^2^. We thus fix the mid-IR peak intensity at 90 GW/cm^2^, corresponding to a vacuum electric peak field amplitude of 0.083 V/Å, and record the optical response of the material as a function of temperature.

Fig. 2*C* shows the results of this measurement. While we find that the fundamental (red dots) does not vary significantly with temperature, all the harmonic orders exhibit varying and strong trends with temperature. Most strikingly, HH7 increases in amplitude by over one order of magnitude over the measured temperature range, while HH3 and HH5 amplitudes initially decrease with temperature. For instance, the amplitude of HH3 decreases until $T^{*}$ = 173 K, after which the amplitude varies very little. A similar trend is observed for HH5. In stark contrast, HH7 increases by at least a factor of 5 until reaching the critical point. This critical point marks the transition between strange metal into the pseudogap phase, at $T^{*}$ = 173 K, and the amplitude of all harmonics varies very little. After further cooling to $T_{\text{c}}$ = 88 K, a dramatic exponential increase is measured for all harmonic orders. This marks the quantum phase transition into the superconducting state. It is interesting to note that the highest nonlinear measure, HH7, is the most sensitive indication of all critical points to investigate the quantum phase transitions from strange metal into pseudogap and SC phases. We note that measurements of the magnetization of the sample readily confirm the temperature value at which the SC phase transition occurs (*SI Appendix*). However, in contrast to HHS, the identification of the transition between strange metal and pseudogap quantum phases is not as obvious from conventional methods like a transport measurement (*SI Appendix*, Fig. S2).

To further elucidate the connection between measured optical nonlinearity and the formation of Cooper pairs in the superconductor, we have developed a theoretical approach that describes HHG in a high-$T_{\text{c}}$ superconductor through a two-band quasi-Hubbard model with BCS d-wave pairing. From the theoretical perspective, high-$T_{\text{c}}$ superconductivity is often described in terms of the Hubbard model (43), a paradigmatic framework in strongly correlated quantum systems capturing both electronic and spin interactions. There have been many theoretical approximations and extensions of the Hubbard model to study the cause of high-$T_{\text{c}}$ superconductivity (44–46). In the literature, high-$T_{\text{c}}$ superconductivity is often modeled with intraorbital and interorbital hopping and electron–electron interactions by a three-band Hubbard model (47, 48). Due to numerical challenges, simplified one-band versions have been considered (45). However, such a simple model does not actively account for the hopping between oxygen orbitals in a nonequilibrium scenario. In contrast, our two-band quasi-Hubbard model uses a band structure calculated from density functional theory, combined with attractive Hubbard-like interactions in the mean-field limit. The electrons in the lower band interact with each other and can therefore be well described by an attractive Hubbard interaction, giving rise to the Cooper pairing and superconductivity. Transitions between the higher band and lower band are to describe high-energy electron hopping processes between the oxygen orbitals. We assume that electrons in the higher band are noninteracting, and dipolar coupling includes intraband and interband transitions. The dynamic evolution of the system is described by semiconductor Bloch equations, including electron–electron and electron–phonon scattering processes through phenomenological dephasing terms; see *SI Appendix* for details on the model.

The calculated high harmonic spectra are shown in Fig. 2*C* (solid lines). We find an excellent match with the experimental data. The model faithfully reproduces the functional form of the measurement data over the entire temperature range and for several orders of magnitude of harmonic amplitude. Most importantly, the model describes the position of critical points, changes of slopes, and the position and trend of exponential harmonic amplitude. The excellent match of simulations with data permits identification of the critical point at $T^{*}$ = 173 K as the transition from the strange metal phase into the pseudogap phase, thus marking the beginning of the fluctuation regime. Microscopically, the transition to the pseudogap phase, by lowering the temperature, is accompanied by strong fluctuations associated with a quantum critical point (49) which is clearly identified by our model and the measurement (Fig. 2*C*). Lowering the temperature further, we expect a further increase in carrier scattering time and a respective increase of the current.

**Fig. 3.**
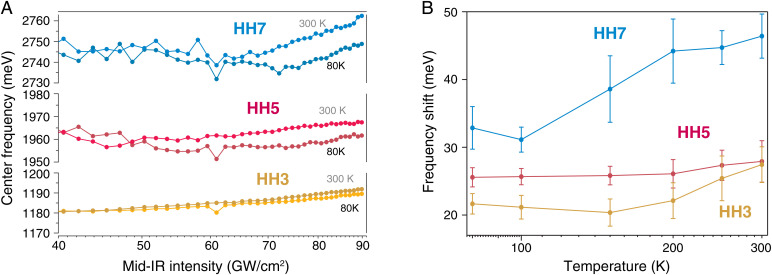
Harmonic frequency and shift: (*A*) The center frequencies of the third, fifth, and seventh harmonic peaks are plotted vs. the intensity of the exciting light at 300 and 80 K. A frequency blueshift increasing with the driving intensity is observed. (*B*) The temperature dependence of the blueshift is shown. All harmonics show a rise of the blueshift for temperatures $T≳T^{*}$. This behavior reflects the suppression of scattering processes in the superconducting phase and the pseudogap phase.

To this end, we extract the blueshift of HHS across all the three regimes: superconducting, pseudogap, and strange metal. The blueshift of HHS is reminiscent of strong electron–electron scattering during HHG (10) and occurs due to a back action of the quasiparticle dynamics on the laser field. The strong carrier scattering and consequential blueshifting are reminiscent of effective energy loss and dephasing of the HHG process. As such, it can reveal crucial details about the quasiparticles in each strongly correlated regime. Fig. 3 shows measurement results which show that at room temperature, carrier scattering is strongest, and the highest nonlinearity, here HH7, is affected most; also see Fig. 1*B*. Interestingly, the directly measurable blueshift, while clearly discernible in all harmonic orders, does allow us to clearly identify the critical temperature and transition between the phases. By comparing Figs. 2 and 3, within the sensitivity of our measurement, the harmonic amplitudes prove to be the strongest observable for carrier scattering.

**Fig. 4.**
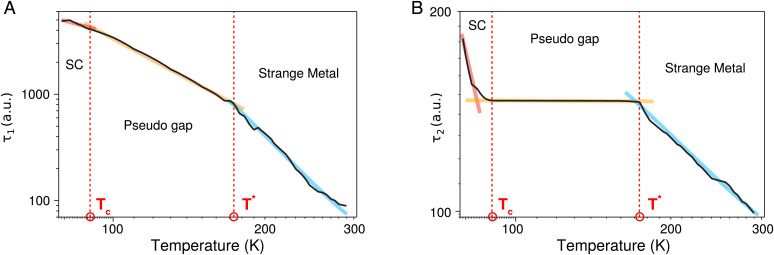
Phenomenological scattering parameters: By fitting the experimental data to the model, we extract the temperature dependence of the phenomenological scattering and dephasing times, shown in atomic units (a.u.), (*A*) *τ*_1_ and (*B*) *τ*_2_. Clearly visible are changes in the functional behavior of the two parameters at the two critical points. In the given temperature range from 80 to 300 K, the scattering time *τ*_1_ decreases from 1400 to 100 a.u. (34 to 2.4 fs), and the dephasing time *τ*_2_ decreases from 180 to 100 a.u. (4.4 to 2.4 fs).

Our model provides insight into this dynamics with phenomenological scattering and dephasing times *τ*_1_ and *τ*_2_, shown in Fig. 4. These two parameters are given by the time scales at which the band population and the correlation functions relax back to their equilibrium value after an excitation (cf. *SI Appendix*, Eq. 2). We note that strong field excitation as in HHS precludes the straightforward interpretation of *τ*_1_ and *τ*_2_ with inverse resistivity. This would be straightforward in the linear excitation regime, but the association with a resistivity under strong-field excitation requires further theoretical development. Nevertheless, both values *τ*_1_ and *τ*_2_ allow identifying the quantum critical points.

Lowering the temperature to 88 K, a striking change of high harmonic amplitude and an exponential increase is observed. This changeover clearly marks the quantum phase transition into the superconducting phase. The theoretical model reproduces the exponential change of HH7 and HH5 in addition to the overall functional form of the experimental curve, and it identifies the dramatic increase in harmonic amplitude due to a vastly increased current from interband carrier motion at near-zero resistivity. We note that a detailed analysis based on an extended microscopic model that could relate the self-energy, due to scattering processes such as electron–electron, electron–phonon, etc., with the measured blueshift would provide a more quantitative picture of the transition from a pseudogap to a non-Fermi liquid phase, i.e., the strange metal phase of the cuprates. The quasi-particle weight extracted from the real part of the self-energy could be used to understand whether a system is in a Fermi liquid state or not. This distinction should be possible because in a Fermi liquid state, the quasi-particle weight is expected to have a finite value at zero temperature, whereas it is zero for a non-Fermi liquid at zero temperature. A large reduction of the quasi-particle weight inferred from the blueshift would reveal the physics of the transition toward the non-Fermi liquid strange metallic phase.

## Conclusions

Our experimental and theoretical findings provide a first striking example of how HHS can be used to distinguish strongly correlated phases of matter and pave the way toward a refined understanding of the intriguing physical processes at work in a high-*T_c_* superconductor. HHS is a technique that is simple to apply but needs a theoretical model to provide microscopic insight. We have provided such a strong-field quasi-Hubbard model which connects the optical spectrum as observable with the microscopic dynamics of carriers. The model describes the formation of composite bosons from fermion spin-1/2 particles by Cooper pairing and the electron hopping processes between orbitals of the material. The combination between experiment and theory permits extracting phenomenological scattering times which clearly identify the quantum critical points. We note that in the linear response regime, these phenomenological amplitudes and phase decay times are directly related to the material’s conductivity. Our BCS-type model faithfully models the measured HHS which is shown to be sensitive to all quantum critical points. Our investigation provides a strong-field investigation of a high $T_{\text{c}}$ superconductor and measurement of its quantum phase transitions. The method clearly shows the efficacy to identify the different quantum phases, such as the pseudogap phase, the strange metal, and the superconducting phase, together with their quantum critical points. We believe that attosecond technology applied to quantum materials provides a view of the multibody physics of strongly correlated materials, and it allows us to probe the pairing glue of Cooper pairs. Such insight will further our understanding of the strong-field and light-field control of light–matter hybrids with the distinct possibility to control competing charge orders in quantum matter. We envision leveraging strong-field control to enable or inhibit superconductivity and to switch quantum phases at the frequency of light. This has implications to develop new ways for energy-efficient information processing and optical quantum computation.

## Supplementary Material

Supplementary File

## Data Availability

All study data are included in the article and/or *SI Appendix*.
