# Supplementary material for: High-harmonic spectroscopy of quantum phase transitions in a high-Tc superconductor
Source: Proc Natl Acad Sci U S A. 2022 Sep 26;119(40):e2207766119. doi: 10.1073/pnas.2207766119 (PMC9546568; doi:10.1073/pnas.2207766119)
Supplement: Supplementary File [file pnas.2207766119.sapp.pdf]

Supporting Information for

High harmonic spectroscopy of quantum phase transitions in a high-T_c superconductor

Jordi Alcalà, Utso Bhattacharya, Jens Biegert, Marcelo Ciappina, Ugaitz Elu, Tobias Graß, Piotr T. Grochowski, Maciej Lewenstein, Anna Palau, Themistoklis P. H. Sidiropoulos, Tobias Steinl, and Igor Tyulnev

Jens Biegert.
E-mail: jens.biegert@icfo.eu

This PDF file includes:

Figs. S1 to S4
SI References

Theoretical model. The theoretical description is based on a Hubbard-like two-band model in the BCS limit, whose time-dependent Hamiltonian reads

$$\begin{aligned} H(t) \;=\; & \sum_{k,\lambda,\sigma} \varepsilon_{k\lambda} c^{\dagger}_{k\sigma\lambda}(t) c_{k\sigma\lambda}(t) + \sum_{k} [\Delta_k(t) c^{\dagger}_{k\uparrow \mathrm{L}}(t) c^{\dagger}_{-k\downarrow \mathrm{L}}(t) + \text{h.c.}] \\ & - E(t) \sum_{k,\lambda,\lambda',\sigma} [c^{\dagger}_{k\sigma\lambda}(t) D_{\lambda\lambda'}(k) c_{k\sigma\lambda'}(t)]. \end{aligned} \qquad [1]$$

Here, $\lambda \in \{\mathrm{L}, \mathrm{U}\}$ is the band index for the lower and upper band, $\sigma \in \{\downarrow, \uparrow\}$ is the spin index, and k is the index for pseudomomentum. The operators $c_{k\sigma\lambda}(t)$ and $c^{\dagger}_{k\sigma\lambda}(t)$ are the corresponding time-dependent annihilation and creation operators in the Heisenberg frame. The band energies are given by $\varepsilon_{k\mathrm{U}} = E_g + E_{\mathrm{U}}(k) - \mu$ and $\varepsilon_{k\mathrm{L}} = E_{\mathrm{L}}(k) - \mu$, where μ is the chemical potential, determined self-consistently. The renormalized band structure along the main symmetry direction is obtained from DFT, and parametrized by the functions $E_\lambda(k)$, and a direct energy gap $E_g = 0.0317$ a.u. at the band edge.

The second term in Eq. (1) is the pairing term, proportional to the time-dependent superconducting gap $\Delta_k(t)$. This term is obtained from an attractive d-wave interaction $U_k = U[\cos(k_x a) - \cos(k_y a)]$ of strength U, and the gap parameter must fulfill the BCS self-consistency condition $\Delta_k(t) = -U_k \left[\sum_{k'} U_{k'} \langle c_{-k'\downarrow \mathrm{L}}(t) c_{k'\uparrow \mathrm{L}}(t)\rangle\right]$.

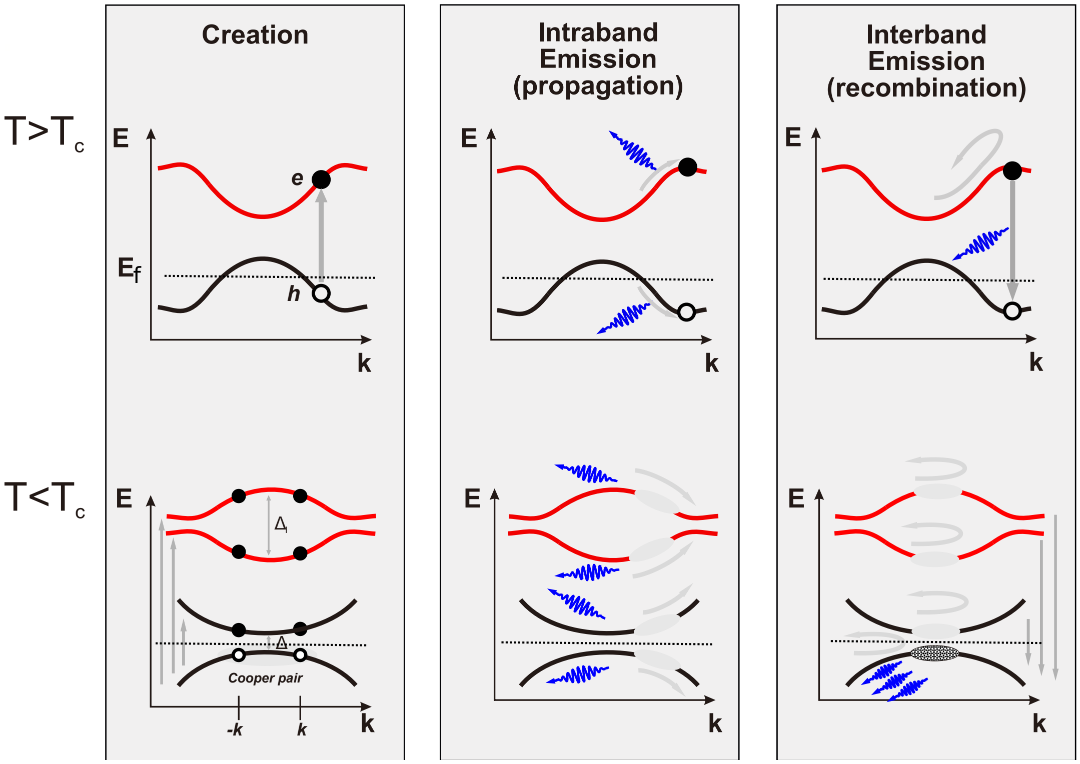

Fig. S1. Three-step model of high harmonic generation: Above T_c (upper line), excitation process, subsequent intraband acceleration, and final interband recombination occur in two bands in vicinity of the Fermi energy E_f. Below T_c (lower line), the superconducting pairing opens a gap at the Fermi energy. This splits the effective band structure of the Cooper pairs, underlying the three processes of high harmonic generation in the strongly correlated phase. Note that in our model, the pairing in the lower band is an equilibrium property of the system, while the pairing in the upper band is induced optically.

The last term in Eq. (1) describes the coupling to the light field, with an electric field pulse $E(t) = E_0 \sin^2(\omega_0 t / 2n_{\mathrm{cyc}}) \sin(\omega_0 t)$ of amplitude $E_0 = 0.004$ in atomic units (a.u.), center frequency $\omega_0 = 0.01425$ a.u., $n_{\mathrm{cyc}} = 8$ cycles. These values are compatible with the experimental estimated parameters of the laser pulse. The optical coupling is proportional to the covariant derivative (1), $-iD_{\lambda\lambda'}(k) = \left[\delta_{\lambda\lambda'}\partial_k - i d_{\lambda\lambda'(k)}\right]$, with $d_{\lambda\lambda'}(k)$ describing the interband dipole moment, or, for $\lambda = \lambda'$, the Berry connection of the respective band, which is zero in our system. For the dipole moment we have used d_{UL}=14 a.u. in our numerical evaluations.

For linearly polarized driving fields, typically one spatial dimension in the k-space is enough to model the electron-hole dynamics (2). As such, we restrict our numerical simulations to only a single one-dimensional slice of the full k-space. From Eq. (1), we then derive the Heisenberg equations of motion which describe the dynamics of the relevant correlators, namely populations $n_{k\lambda\sigma}(t) = \langle c^{\dagger}_{k\sigma\lambda}(t) c_{k\sigma\lambda}(t)\rangle$, normal interband polarizations $P^{*}_{k\downarrow}(t) = \langle c^{\dagger}_{k\downarrow\mathrm{U}}(t) c_{k\downarrow\mathrm{L}}(t)\rangle$ and $P^{*}_{k\uparrow}(t) = \langle c^{\dagger}_{k\uparrow\mathrm{U}}(t) c_{k\uparrow\mathrm{L}}(t)\rangle$, as well as the anomalous mixed-spin polarizations $A_{k\uparrow\downarrow}(t) = \langle c^{\dagger}_{k\uparrow\mathrm{U}}(t) c^{\dagger}_{-k\downarrow\mathrm{L}}(t)\rangle$ and $A_{k\downarrow\uparrow}(t) = \langle c^{\dagger}_{k\downarrow\mathrm{U}}(t) c^{\dagger}_{-k\uparrow\mathrm{L}}(t)\rangle$, and the superconducting correlations $S_{k\mathrm{U}}(t) = \langle c^{\dagger}_{k\uparrow\mathrm{U}}(t) c^{\dagger}_{-k\downarrow\mathrm{U}}(t)\rangle$ and $S_{k\mathrm{L}}(t) = \langle c^{\dagger}_{k\uparrow\mathrm{L}}(t) c^{\dagger}_{-k\downarrow\mathrm{L}}(t)\rangle$. These "superconductor Bloch equations" form a closed set of first-order differential equations. In order to account also for effects due to electron-electron and

electron-phonon interactions and disorder, we include in these equations non-unitary scattering processes with phenomenological scattering times τ_1 and τ_2:

$$i\partial_t\langle O(t)\rangle = -\langle[H(t), O(t)]\rangle - i\frac{\langle O(t)\rangle}{\tau_{1/2}}. \qquad [2]$$

τ_1 is used when $O(t)$ represents a population, while τ_2 when $O(t)$ describes a correlation. The scattering times τ_1 and τ_2 and their temperature-dependence are obtained by fitting theoretical results to the experimental data, see main text Fig. 4.

The initial conditions of the equations of motion are given by the thermal equilibrium when the electric field is off. The band populations are given by the Fermi-Dirac distribution, assuming a half-filled L-band. All polarizations and the superconducting correlator in the U-band are initially zero. The superconducting correlator in the L-band takes nonzero values up to the critical temperature. Its value is determined self-consistently by solving the model through the Bogoliubov-de Gennes transformation. With the phenomenologically motivated choice of $U = 0.718$ and a half-filled L-band, the model produces the right critical temperature $T_\mathrm{c} = 88$ K.

We then time-evolve the system in the optical field, and evaluate the generated electrical current J in the crystal (1), given by $J(t) = J_\mathrm{intra}(t) + J_\mathrm{inter}(t)$ with $J_\mathrm{intra} = \sum_{k,\lambda,\sigma} \partial_k \varepsilon_{k\lambda} \langle n_{k\lambda\sigma}\rangle$ and $J_\mathrm{inter} = \sum_{k\sigma} 2\, d_\mathrm{UL}\,(\varepsilon_{k\mathrm{U}} - \varepsilon_{k\mathrm{L}})\,\mathrm{Im}(P_{k\sigma})$, assuming a constant interband dipole moment d_UL. Here, we have already assumed a one-dimensional geometry. From the time-dependent current $J(t)$ we obtain the harmonic spectrum by Fourier transforming it into the frequency domain, with the results shown in Fig. 2(c) of the main text.

A pictorial illustration of the high harmonic generation, from the point of view of a three-step-model, is given in Fig. S1.

YBCO band structure. The band structure shown in Fig. S2 has been calculated with the all-electron full-potential linearised augmented-plane wave (LAPW) code ELK (3). For simplicity, the Γ–X–S direction is used for the simulations.

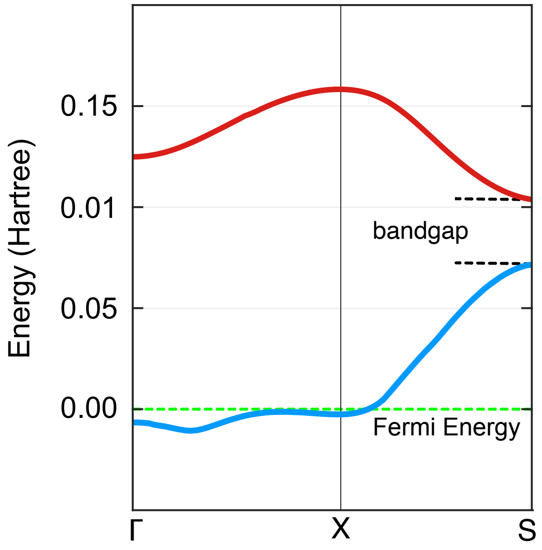

Fig. S2. Band structure: The two bands U and L are plotted along Γ–X–S. The horizontal line marks the Fermi energy for a half-filled L-Band.

High Harmonic Spectroscopy and Data Analysis. The third (1065 nm), fifth (639 nm) and seventh (457 nm) harmonics generated driven by the 3.2 μm pulses are measured with a custom configured Maya 2000 Pro high-sensitivity spectrometer from Ocean Insight, Inc. The generated harmonics at the surface of the YBCO sample are imaged in free space onto a 600–μm–wide spectrometer slit. The spectrum of all three harmonics is measured simultaneous using an integration time of 500 ms. We used a FGB37 bandpass filter from Thorlabs, Inc to measure all the signals together. The purpose of the FGB37 bandpass filter is a calibrated attenuation of the third harmonic signal by ~ 4 orders of magnitude such that HH3 can be measured together with the much weaker HH5 and HH7. The filter avoids saturation and possible cross-talk between the array pixels and readout. The high dynamic range of the Maya 2000 Pro spectrometer enables measuring HH5 and HH7 signals without requiring any additional attenuation of HH5. We have conducted additional measurements with a monochromator and calibrated photomultiplier tube to confirm the accuracy of our measurements. The spectrometer's integration time was set to 500 ms to minimise thermal effects during measurement. In addition, an automatic shutter blocked the mid-IR beam for a few minutes between each measurement to ensure thermalisation of the sample to the set temperature before another acquisition was taken. The YBCO temperature was varied from cryogenic to room temperatures and back to avoid systematic errors due to possible hysteresis. This process was repeated numerous times. The standard deviation is calculated from those repeated measurements.

Scaling of the reflected fundamental field with temperature. For completeness, Fig. S3 displays the data for the fundamental field, shown in Fig. 2(c), on a linear intensity scale.

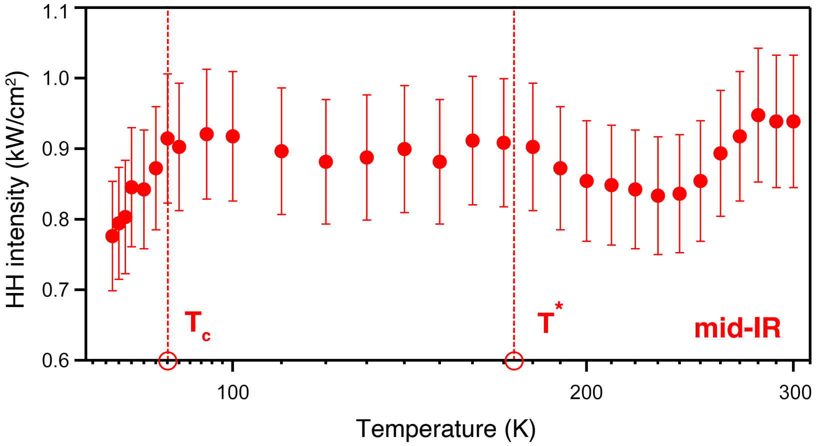

Fig. S3. Reflectivity of the fundamental field on YBCO: Shown is the reflected mid-IR field as a function of temperature on a linear intensity scale. The data is the one shown in Fig. 2(c). These measurements are taken for a mid-IR field strength of 0.083 V/Å.

YBCO sample fabrication and characterisation. $YBa_2Cu_3O_{7-d}$ (YBCO) films with a thickness of 100 nm were grown using pulsed laser deposition (PLD) on 5×5 mm^2 $LaAlO_3$ (100) single crystal substrates with thicknesses of 500 μm. The films where deposited at 800°C and PO_2 = 0.3 mbar with a pulse frequency of 5 Hz and oxygenated at 600°C at 1 bar. After film growth the substrate was milled to a final thickness of 50 μm. SQUID magnetometry (Quantum Design) was used to determine the critical current temperature and critical current density using the Bean critical state model. Transport measurements were performed with four-probe configuration by a commercial physical property measurement systems (PPMS). The results from these measurements are shown in Fig. S4. We note that these measurements are in very good agreement with literature values; see e.g. Ref. (4).

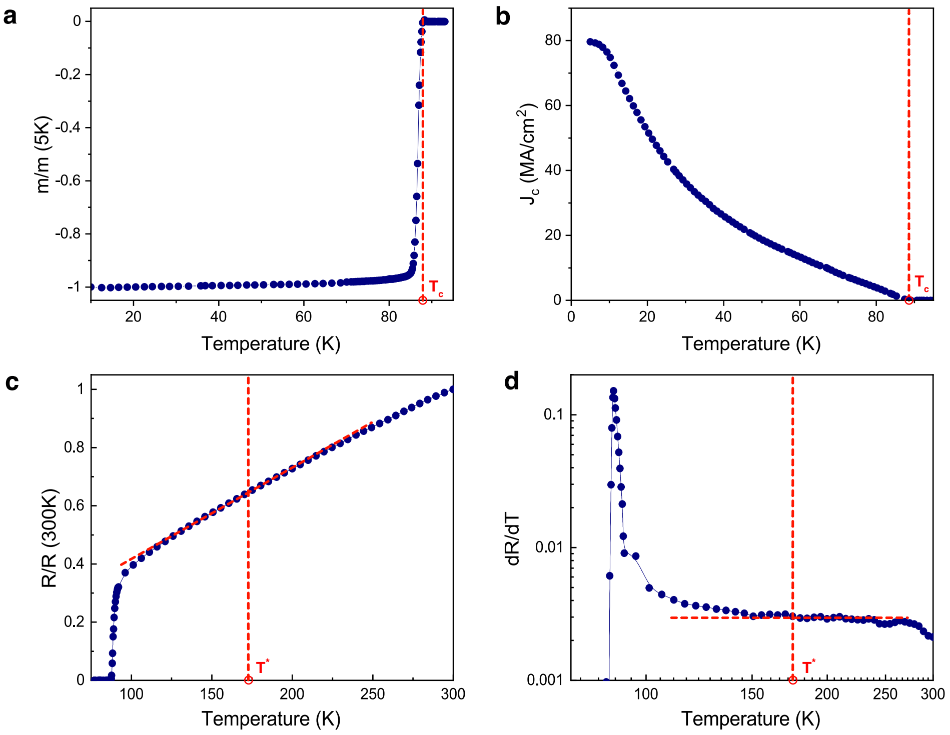

Fig. S4. Characterization of YBCO samples: (a) SQUID measurement of the temperature dependence of the magnetic moment, normalised at 5 K, showing a critical temperature ofT_c = 88 K and (b) critical current density as a function of temperature, J_c(T). (c) Shows the resistivity, normalised at 300 K, as function of temperature and (d) shows the derivative. For completeness the HHS determined critical temperatures are superimposed. Solid lines in (c) and (d) are linear fits performed above T^*.

References

1. GB Ventura, DJ Passos, JMB Lopes dos Santos, JM Viana Parente Lopes, NMR Peres, Gauge covariances and nonlinear optical responses. *Phys. Rev. B* **96**, 035431 (2017).
2. G Vampa, et al., Theoretical analysis of high-harmonic generation in solids. *Phys. Rev. Lett.* **113**, 073901 (2014).
3. The Elk Code (http://elk.sourceforge.net/) (2022).
4. L Taillefer, Scattering and Pairing in Cuprate Superconductors. *Annu. Rev. Condens. Matter Phys.* **1**, 51–70 (2010).
